# Deaths in Philadelphia from April 20th to June 22d, 1850

**Published:** 1850-07

**Authors:** James Aitken Meigs

**Affiliations:** Student of Medicine


*Deaths in Philadelphia from April 20th to June 22d,* 1850. Reported by Mr. JAMES AITKEN MEIGS, Student of Medicine.

| Diseases. | Ad'ts | Chil. | Diseases. | Ad'ts | Chil. |
|---|---|---|---|---|---|
| Abscess, . . . | 3 | 0 | Fever, puerperal, . . | 5 | 0 |
| " of lungs . . | 1 | 1 | " remittent, . . | 1 | 2 |
| " of throat, . . | 0 | 1 | " scarlet, . . | 3 | 103 |
| Anæmia, . . . | 4 | 1 | " typhoid, . . | 9 | 3 |
| Aneurism of Heart, . . | 1 | 0 | " typhus, . . | 8 | 4 |
| Angina Pectoris, . . | 0 | 1 | Fracture, . . . | 1 | 0 |
| Apoplexy, . . . | 20 | 0 | " of skull, . . | 1 | 0 |
| " Pulmonary, . | 1 | 0 | Fungus hæmatodes, . | 1 | 0 |
| Asthma, . . . . | 2 | 0 | Gangrene of leg, . . | 1 | 0 |
| Burns and Scalds, . . | 3 | 8 | Gout, . . . . | 1 | 0 |
| Cachexia, . . . | 1 | 0 | Gun-shot wound, . . | 0 | 1 |
| Cancer of breast, . . | 4 | 0 | Hemorrhage, . . . | 2 | 1 |
| " stomach, . | 2 | 0 | " from uterus, . | 2 | 0 |
| " uterus, . . | 2 | 0 | Hæmoptysis, . . . | 7 | 1 |
| Cancrum oris, . . | 0 | 4 | Hæmatemesis, . . | 1 | 0 |
| Caries of spine, . . | 0 | 1 | Hernia, . . . . | 1 | 2 |
| Casualties, . . . | 2 | 5 | Hydrocephalus, . . | 0 | 61 |
| Cholera infantum, . . | 0 | 29 | Hydropericardium, . . | 6 | 1 |
| " morbus, . . | 4 | 1 | Hydrothorax, . . . | 9 | 9 |
| Colic, . . . . | 1 | 0 | Inanition, . . . | 1 | 3 |
| Compression of brain, . | 0 | 1 | Inflammation of brain, . | 5 | 27 |
| Congestion of lungs, . | 4 | 4 | " breast, . | 0 | 1 |
| " brain, . | 7 | 12 | " bronchi, . . | 6 | 28 |
| Convulsions, . . . | 2 | 74 | " diaphragm, . | 1 | 0 |
| " puerperal, . | 1 | 0 | " heart, . . | 2 | 0 |
| Croup, . . . . | 0 | 22 | " kidneys, . . | 1 | 0 |
| Cyanosis, . . . | 0 | 3 | " larynx, . . | 1 | 2 |
| Debility, . . . | 9 | 37 | " liver, . . | 8 | 0 |
| Diarrhœa, . . . | 7 | 13 | " lungs, . . | 22 | 55 |
| Disease of brain, . . | 4 | 17 | " peritoneum, . | 5 | 5 |
| " chest, . . | 2 | 0 | " pleura, . . | 2 | 0 |
| " heart, . . | 10 | 7 | " stom. & bowels, | 15 | 22 |
| " liver, . . | 3 | 0 | " uterus, . . | 0 | 1 |
| " lungs, . . | 0 | 4 | Intemperance, . . | 11 | 0 |
| " ovary, . . | 1 | 0 | Jaundice, . . . | 0 | 3 |
| " spleen, . . | 0 | 1 | Malformation, . . | 0 | 2 |
| " stomach and bowels, | 1 | 1 | " of heart, . | 0 | 2 |
| Dropsy, . . . | 11 | 4 | Mania, . . . . | 2 | 0 |
| " abdominal, . | 1 | 1 | Mania-a-potu, . . | 10 | 0 |
| Drowned, . . . | 13 | 9 | Marasmus, . . . | 1 | 18 |
| Dysentery, . . . | 9 | 23 | Measles, . . . | 0 | 27 |
| Dyspepsia, . . . | 1 | 0 | Melanosis, . . . | 1 | 0 |
| Effusion on brain, . . | 2 | 11 | Mortification, . . . | 0 | 1 |
| Emphysema pulmonary, | 1 | 2 | " of mouth & throat, | 0 | 2 |
| Erysipelas, . . . | 8 | 7 | Neuralgia, . . . | 1 | 0 |
| Fever, . . . . | 0 | 6 | Old age, . . . . | 29 | 0 |
| " congestive, . . | 0 | 1 | Obstruction of intestines, | 0 | 1 |
| " continued, . . | 1 | 0 | Parturition, . . . | 2 | 0 |
| " intermittent, . | 1 | 1 | Palsy, . . . . | 7 | 1 |

| Diseases. | Ad'ts | Chil. | Diseases. | Ad'ts | Chil. |
|---|---|---|---|---|---|
| Phlegmasia dolens, | 1 | 0 | Sunstroke, | 1 | 0 |
| Phthisis pulmonalis, | 138 | 32 | Syncope, | 1 | 0 |
| Pertussis, | 0 | 10 | Syphilis, | 1 | 0 |
| Poisoning, | 4 | 3 | Tabes mesenterica, | 0 | 4 |
| Purpura, | 1 | 1 | Teething, | 0 | 3 |
| Rheumatism, | 2 | 0 | Tetanus, | 2 | 0 |
| Scirrhus of stomach, | 1 | 0 | Ulceration of liver, | 1 | 0 |
| Scrofula, | 3 | 10 | " throat, | 0 | 4 |
| Small pox, | 1 | 5 | " bowels, | 4 | 1 |
| Softening of stomach, | 0 | 1 | Unknown, | 12 | 14 |
| " brain, | 1 | 1 | Violence, | 1 | 1 |
| Sphacelus of throat, | 0 | 1 | Varicella, | 0 | 1 |
| Still born, | 0 | 86 | Wounds, | 0 | 1 |
| Suffocation, | 1 | 1 | | | |
| Suicide, | 1 | 0 | | 519 | 882 |
| Total, | | | | 1401 | |

Of the foregoing the ages were as follows:—

| | | | |
|---|---|---|---|
| Under | 1 year, | | 345 |
| From | 1 | to 2, | 163 |
| | 2 | 5, | 222 |
| | 5 | 10, | 81 |
| | 10 | 15, | 26 |
| | 15 | 20, | 43 |
| | 20 | 30, | 124 |
| | 30 | 40, | 111 |
| | 40 | 50, | 97 |
| | 50 | 60, | 57 |
| | 60 | 70, | 50 |
| | 70 | 80, | 43 |
| | 80 | 90, | 29 |
| | 90 | 100, | 10 |
| | | | 1401 |

Included in this number, are 95 from the Almshouse, 121 people of color, and 14 from the surrounding country.

